# Cardiac Autonomic Neuropathy in Diabetic Peripheral Neuropathy

**DOI:** 10.7759/cureus.67777

**Published:** 2024-08-25

**Authors:** Balaji Naik, Abhishek Pandey, Riddhi Dasgupta, Channabasappa Shivaprasad

**Affiliations:** 1 Diabetes and Endocrinology, Sapthagiri Institute of Medical Sciences and Research Centre, Bengaluru, IND; 2 Medicine, Institute of Medical Sciences, Banaras Hindu University, Varanasi, IND

**Keywords:** diabetic microvascular complications, sympathetic nervous system, parasympathetic nervous system, diabetic peripheral neuropathy, cardiac autonomic neuropathy

## Abstract

Background

Cardiac autonomic neuropathy (CAN) is the most underdiagnosed consequence of diabetes because standard hospital settings do not provide consistent diagnostic criteria or testing resources. It is still unclear how diabetic peripheral neuropathy (DPN) and CAN are related. Therefore, this study aimed to determine the prevalence of CAN in individuals with type 2 diabetes mellitus who had isolated DPN without other microvascular or macrovascular complications.

Methodology

A total of 35 type 2 diabetes mellitus patients with isolated DPN (group 1) and an equal number of sex- and age-matched patients without DPN (group 2) underwent CAN testing. Results were compared between the two groups.

Results

A significantly higher prevalence of isolated parasympathetic (28.57 vs. 11.42%), isolated sympathetic (22.85 vs. 8.57%), and combined autonomic dysfunction (37.14 vs. 2.85%) was found in the neuropathic group compared to the non-neuropathic group. Group 1 exhibited more abnormal parasympathetic nervous system test results and increased diastolic pressure during sustained handgrip compared to group 2 (all p-values <0.05).

Conclusions

A significantly higher prevalence of cardiac autonomic dysfunction is seen in patients with DPN without other microvascular or macrovascular complications, irrespective of age, sex, or duration of diabetes mellitus. Patients with a higher body mass index were found to have significantly increased cardiac autonomic dysfunction.

## Introduction

Neuropathy, both peripheral and autonomic, is a prevalent complication of diabetes mellitus [1]. Diabetic peripheral neuropathy (DPN) is a chronic, disabling condition that frequently results in pain, tingling sensation, foot ulceration, frequent falls, and lower limb amputations. One of the most common consequences of diabetes mellitus that is not well recognized and underdiagnosed is cardiac autonomic neuropathy (CAN) [2]. It is an independent cardiovascular morbidity risk factor. Orthostatic hypotension, resting tachycardia, effort intolerance, and intraoperative cardiovascular liability are among the clinical symptoms of CAN [3]. The pathophysiology of CAN is complex, multifactorial, and unclear. Numerous pathogenic mechanisms, including the formation of advanced glycation end products, increased oxidative stress with increased production of free radicals, activation of the polyol and protein kinase C pathways, and activation of polyADP ribosylation are implicated in autonomic nervous system dysfunction in diabetic patients. In addition, its prevalence increases with the duration of diabetes in both type 1 and type 2 diabetes mellitus [4,5]. Various studies have demonstrated variable prevalence in individuals with type 2 diabetes mellitus with multiple microvascular and macrovascular problems due to the use of diverse criteria for the diagnosis of CAN [6]. It remains underdiagnosed due to a lack of uniform diagnostic criteria and testing resources in typical hospital settings [6]. Considering that cardiovascular denervation is partly reversible in the early stages and its development and progression can be mitigated, early screening for CAN is crucial for preventing serious complications and mortality associated with it. CAN advancement is linked to microvascular problems such as nephropathy and retinopathy secondary to diabetes; however, a clear relationship between DPN and CAN is yet to be established [7]. While some studies have shown an increased prevalence of cardiac autonomic dysfunction in patients with painful DPN, limited information is available from India regarding the precise frequency and pattern of CAN in isolated DPN patients irrespective of pain [8]. Evidence for isolated DPN as an early indicator of CAN development and progression in patients with type 2 diabetes mellitus and no other complications is limited. Therefore, in this study, we aimed to estimate the prevalence of CAN in patients with type 2 diabetes mellitus and isolated DPN and no other microvascular or macrovascular complications. Our findings will help in the early diagnosis and aggressive management of CAN to prevent its associated morbidity and mortality.

## Materials and methods

Study design

**Figure 1:**
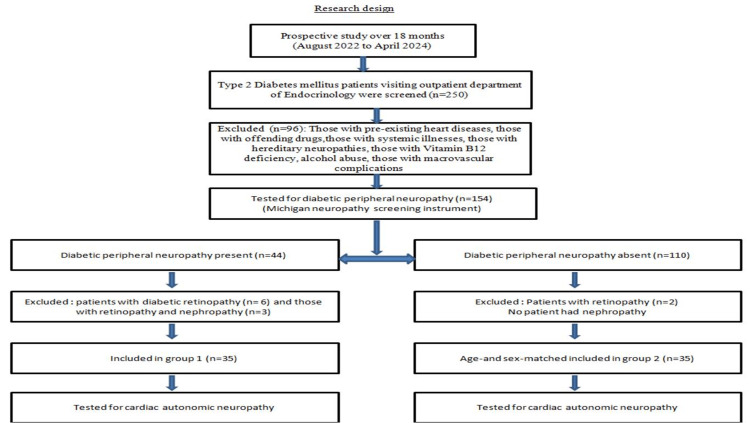
Flowchart detailing the study design.

This prospective study included 250 patients with type 2 diabetes mellitus who visited our outpatient endocrinology department at Sapthagiri Institute of Medical Sciences and Research Center, Bengaluru, India, between August 2022 and April 2024. Inclusion criteria were as follows: (1) age 35 years or older; and (2) a type 2 diabetes mellitus diagnosis, as specified by the American Diabetes Association standards: fasting plasma glucose ≥126 mg/dL, two-hour plasma glucose ≥ 00 mg/dL or HbA1C ≥6.5%, and plasma glucose ≥200 mg/dL during hyperglycemic symptoms or crisis [9]. Patients with concomitant conditions that can affect cardiac autonomic functions (such as coronary artery disease, rheumatic heart disease, cardiac arrhythmias, and congestive cardiac failure), those receiving drugs known to affect cardiac autonomic function (such as neurotoxic medications, beta-blockers, sympathomimetics, and antiarrhythmic drugs), and those with conditions that affect the autonomic nervous system (such as thyroid disorders, severe systemic illnesses, malignancy, alcohol abuse, vitamin B12 deficiency, rheumatological conditions, and hereditary forms of neuropathies) were excluded. We obtained informed consent from all participants. The study was approved by the Institutional Ethics Committee of Sapthagiri Institute of Medical Sciences and Research Center, Bengaluru, India (reference number: SIMS&RC/EC/14/2023). We enrolled 70 patients with type 2 diabetes mellitus who were divided into two groups, as depicted in Figure 1.

Study population

We screened 250 patients with type 2 diabetes mellitus. After excluding patients with other macrovascular complications of diabetes mellitus and those meeting the abovementioned exclusion criteria, we selected 154 patients for peripheral neuropathy testing.

Diabetic peripheral neuropathy testing

The Michigan Neuropathy Screening Instrument was utilized in this study to identify DPN [10]. Patients completed a comprehensive questionnaire comprising 15 self-administered “yes” or “no” questions regarding neuropathic symptoms. In addition, they underwent a thorough physical examination, which included a monofilament test with appropriate scoring and an assessment of neuropathy involving inspection of the feet, vibration sensation, and ankle reflexes.

Individuals who scored >/=7 on the questionnaire and ≥2.5 on the physical assessment were classified as having DPN [10]. Patients with DPN were further evaluated for the presence of other microvascular complications, such as diabetic retinopathy or nephropathy. Patients with at least one microvascular complication along with DPN were excluded. The remaining 35 patients were included in Group 1. Group 2 comprised an equal number of age- and sex-matched patients with type 2 diabetes mellitus without DPN or any other microvascular complications (n = 35).

Clinical and biochemical characteristics

A thorough clinical history was obtained, and physical examinations including body mass index (BMI) and blood pressure were performed. Basic biochemical parameters (HbA1C, fasting plasma glucose levels, postprandial plasma glucose levels, fasting lipid profile, renal function tests, and urinary spot microalbumin levels) were recorded.

Cardiac autonomic neuropathy testing

The most reliable method for assessing cardiac autonomic function is cardiac autonomic reflex testing [11]. We evaluated cardiac autonomic function using the KODYSCAN system (Kody Medical Electronics Pvt. Ltd, Chennai, India). The system analyzed both the sympathetic nervous system (SNS) and parasympathetic nervous system (PNS) functions using data from electrocardiogram (ECG) and automatic non-invasive blood pressure measurements to conduct the tests. Per standard protocol, we conducted autonomic function tests during the mornings. Patients were instructed to avoid smoking or drinking coffee at least three hours before testing.

Parasympathetic function testing

Heart rate variability between the longest and shortest RR intervals in response to deep breathing, standing, and the Valsalva maneuver was used to measure parasympathetic functions. The ratio of the RR interval at six breaths per minute during deep breathing was used to compute the response to deep breathing (E/I ratio). On standing, heart rate response was determined by calculating the difference between the RR interval at the 15th and 30th beat using an ECG that recorded the heart rate over two minutes while the subject was supine. Before, during, and one minute after the Valsalva maneuver (Valsalva ratio), the heart rate was monitored. During this process, the longest and shortest RR intervals were used to compute the Valsalva ratio.

Sympathetic function testing

The greatest diastolic blood pressure increase was achieved with a prolonged hand grip, and the postural drop in systolic blood pressure upon standing was used to measure sympathetic function. During the five-minute sustained hand grip test, the patient’s contralateral arm’s automated non-invasive blood pressure was taken while they squeezed a hand grip dynamometer at 30% of its maximal strength. Blood pressure readings were taken while the patient was supine for two minutes, and then again three minutes after the patient stood up to look for any postural drops in systolic blood pressure.

Interpretation of tests

All test findings were classified as normal, borderline, or abnormal based on Ewing’s criteria [12]. E/I ratios ≥1.21 with deep breathing, 30:15 ratios ≥1.04 when standing, and Valsalva ratios ≥1.21 during the Valsalva maneuver were considered normal assessments for heart rate variability. When standing, a fall in systolic blood pressure of at least 10 mmHg and a rise in diastolic blood pressure by at least 16 mmHg with prolonged hand gripping were deemed normal. Results of the test that were considered abnormal included an E/I ratio of less than 1.10, a 30:50 ratio of less than 1.0, a Valsalva ratio of less than 1.10, a drop in systolic blood pressure of more than 30 mmHg when standing, and a rise in diastolic blood pressure of less than 10 mmHg during prolonged handgrip. Values between normal and abnormal were labeled “Borderline.”

Classification based on autonomic function test abnormalities

Autonomic function test results were classified as having PNS, SNS, and combined abnormalities based on the studies by Ewing et al. Patients were defined as having a PNS abnormality if one or more heart rate variability test results were abnormal. If one or more blood pressure response test results were abnormal, the patient was considered to have an SNS abnormality. If any of the following combinations were present, the patient was considered to have combined dysfunction of both the PNS and SNS: (1) a minimum of one abnormal PNS test result in conjunction with a borderline SNS test result; (2) a minimum of one borderline PNS test result in conjunction with an abnormal SNS test result; and (3) any abnormal PNS test result in conjunction with any SNS test result [13].

Statistical analysis

If the data were normally distributed, all continuous variables were displayed as means with standard deviations. Medians with ranges were used to characterize variables having skewed distributions. Frequencies and percentages were used to characterize all other categorical variables. SPSS version 17.0 was used to conduct the statistical analysis (SPSS, Inc., Chicago, IL, USA). Using a one-way analysis of variance (ANOVA), we examined the clinical and biochemical data of patients with normal, abnormal, and borderline PNS and SNS functioning. A two-tailed p-value ≤0.05 was considered statistically significant.

## Results

In the study, we enrolled 70 patients, who were split into two groups of 35 each. Patients with type 2 diabetes mellitus who had DPN comprised group 1, and an equal number of patients who were age- and sex-matched but did not have DPN comprised group 2. We examined the research participants’ biochemical and demographic data. The individuals’ average age ranged from 36 to 79 years, their mean HbA1C level was 8.15%, and their mean duration of diabetes was 9.5 years. The two groups’ mean age, duration of diabetes, HbA1C level, and other characteristics were comparable. Nonetheless, group 1’s mean BMI was noticeably greater than group 2’s. (29.1 vs. 26.1, p = 0.04). Study participants’ demographic and biochemical characteristics are presented in Table 1.

**Table 1:** Study participants’ biochemical and demographic variables. BMI: body mass index; OAD: oral antidiabetic drug; HDL: high-density lipoprotein; LDL: low-density lipoprotein; BP: blood pressure

Variable	Group 1 (n = 35)	Group 2 (n = 35)	P-value (group 1 and group 2)
Age (years)	50.9 ± 11.1	48.8 ± 7.8	0.356
Sex: male	24 (71.4%)	29 (82.9%)	0.255
BMI (kg/m^2^)	29.1 ± 4.4	26.1 ± 4.8	0.046
Duration of diabetes (years)	10 (6, 12)	9 (5, 12)	0.586
OAD	27 (77.14%)	28 (80%)	1.00
Insulin	1 (2.85%)	0	
OAD + insulin	7 (20%)	7 (20%)	
Smoking	11 (31.4%)	10 (28.6%)	0.794
Hemoglobin (g/dL)	12.1 ± 1.99	11.8 ± 1.87	0.449
HbA1C (%)	8.13 ± 1.11	8.85±1.91	0.059
Serum creatinine (mg/dL)	0.97 ±0.20	0.98 ± 0.21	0.764
Total cholesterol (mg/dL)	204.4 ± 51.2	185.1 ± 48.7	0.111
Triglycerides (mg/dL)	212 (150, 300)	158 (127, 235)	0.285
LDL-cholesterol (mg/dL)	133.8 ± 49.6	118.4 ± 54.3	0.219
HDL-cholesterol(mg/dL)	34.9 ± 8.4	41.4 ± 9.1	0.003
Urinary microalbumin (spot)	4 (2, 8)	4 (2, 6)	0.720
Systolic BP (mm Hg)	135.8 ± 21.5	133.2 ± 13.1	0.356
Diastolic BP(mm Hg)	86.9 ± 16.7	87.2 ± 13.2	0.069

Assessment of participants’ cardiac autonomic neuropathy

**Figure 2:**
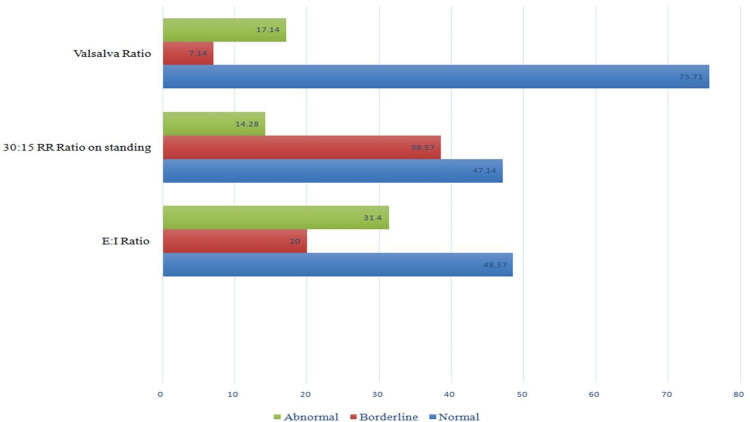
Pattern of parasympathetic nervous system dysfunction in the study population. X-axis: percentage of patients having parasympathetic function test abnormalities; Y-axis: individual parasympathetic function tests.

**Figure 3:**
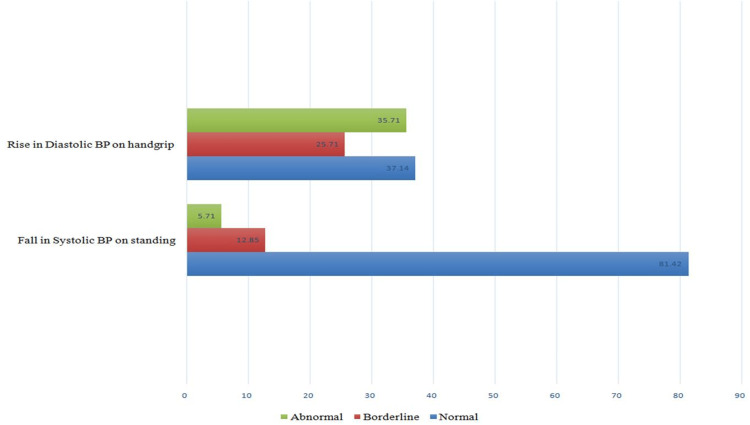
Pattern of sympathetic nervous system dysfunction in the study population. X-axis: percentage of patients having sympathetic function test abnormalities: Y-axis: individual sympathetic function tests. BP: blood pressure

Evaluation of CAN in the study participants revealed that a low E/I ratio was the most prevalent PNS abnormality (31.4%) and increased diastolic pressure during sustained handgrip was the most prevalent SNS abnormality (35.7%). The pattern of parasympathetic nervous system involvement in study participants is depicted in Figure 2. The pattern of sympathetic nervous system involvement is shown in Figure 3.

Pattern of cardiac autonomic dysfunction in individual groups

In group 1, increased diastolic pressure during sustained handgrip (60%) was the most prevalent autonomic and SNS abnormality. In addition, a decreased E/I ratio was the most prevalent PNS abnormality in these patients. In group 2 patients, increased diastolic pressure during sustained handgrip and decreased Valsalva ratio during Valsalva maneuver were the most common autonomic dysfunctions, with each having a prevalence of 11.4%. While increased diastolic pressure during sustained handgrip was the most prevalent SNS abnormality, values of the Valsalva ratio during the Valsalva maneuver were more abnormal than those of group 1. Compared to group 2, group 1 showed more aberrant PNS test results and higher diastolic pressure during sustained handgrip (all p-values <0.05). Parasympathetic and sympathetic nervous system involvement in individual groups is shown in Table 2.

**Table 2:** Cardiac autonomic neuropathy pattern in individual groups.

Parasympathetic nervous system		Group 1, n (%)	Group 2, n (%)	P-value
E/I ratio	Normal	10 (28.57%)	24 (68.57%)	<0.001
	Borderline	6 (17.14%)	8 (22.85%)	
	Abnormal	19 (54.28%)	3 (8.57%)	
30:15 ratio on standing	Normal	11 (31.42%)	22 (62.85%)	0.001
	Borderline	14 (40%)	13 (37.14%)	
	Abnormal	10 (28.57%)	0	
Valsalva ratio	Normal	27 (77.14%)	26 (74.28%)	0.041
	Borderline	0	5 (14.28%)	
	Abnormal	8 (22.85%)	4 (11.42%)	
Sympathetic nervous system		Group 1, n (%)	Group 2, n (%)	P-value
Postural drop in systolic blood pressure on standing	Normal	27 (77.14%)	30 (85.71%)	0.164
	Borderline	4 (11.42%)	5 (14.28%)	
	Abnormal	4 (11.42%)	0	
Rise in diastolic blood pressure on sustained handgrip	Normal	5 (14.28%)	21 (60%)	<0.001
	Borderline	8 (22.85%)	10 (28.57%)	
	Abnormal	21 (60%)	4 (11.42%)	

Classification of overall cardiac autonomic involvement

Of the total 70 patients, 15.71% had isolated SNS dysfunction, 20% had isolated PNS dysfunction, and 20% had combined SNS and PNS dysfunctions. However, 44.28% of patients did not have any cardiac autonomic dysfunction, as shown in Table 3.

**Table 3:** Classification of overall cardiac autonomic involvement. ^a: ^group 1 exhibited considerably more isolated parasympathetic dysfunction than group 2 (p < 0.05); ^b^: group 1 experienced considerably more isolated sympathetic dysfunction than group 2 (p < 0.05); ^c^: group 1 exhibited significantly higher levels of combined sympathetic and parasympathetic dysfunction in comparison to group 2 (p < 0.05).

	No autonomic dysfunction, n (%)	Only parasympathetic dysfunction, n (%)	Only sympathetic dysfunction, n (%)	Combined parasympathetic and sympathetic dysfunction, n (%)	P-value
Overall (n = 70)	31 (44.28%)	14 (20%)	11 (15.71%)	14 (20%)	
Group 1 (n = 35)	4 (11.42%)	10 (28.57%)	8 (22.85%)	13 (37.14%)	<0.001
Group 2 (n = 35)	27 (77.14%)	4 (11.42%)^a^	3 (8.57%)^b^	1 (2.85%)^c^	

In group 1, 28.57%, 22.85%, and 37.14% of patients exhibited isolated PNS, isolated SNS, and combined PNS and SNS abnormalities, respectively. In group 2, 11.42%, 8.57%, and 2.85% of patients exhibited isolated PNS, isolated SNS, and combined PNS and SNS abnormalities, respectively. Compared to group 2, group 1 had greater prevalences of isolated PNS, isolated SNS, and combined PNS and SNS abnormalities (all p-values <0.05).

## Discussion

Our study’s main goal was to determine how common CAN was in DPN patients without any additional micro- or macrovascular complications. The study covered 70 patients in total. Individuals with type 2 diabetes mellitus with DPN comprised group 1, whereas individuals of the same age and sex but without DPN comprised group 2.

Demographic and biochemical parameters

The average age of the patients in group 1 in our investigation was 50 years, which was in line with the findings of a related study by Gaede et al. [14]. The average duration of diabetes was 10 years. Bhuyan et al.’s study, which assessed cardiac autonomic dysfunction in 100 type 2 diabetic patients, found a similar duration [15]. The duration of diabetes is thought to be a separate risk factor for the onset of CAN. Nonetheless, research by Witte et al. demonstrated that cardiac autonomic dysfunction can be identified at the time of diabetes diagnosis [7].

Body mass index

The mean BMI of group 1 in our study was 29 kg/m^2^, which was substantially higher than group 2’s BMI (p < 0.05), indicating that in our community, a higher BMI may be associated with an increased risk of cardiac autonomic dysfunction. Obesity as a contributory factor to CAN was studied by Ziegler et al. and Akhter et al., with findings similar to those observed in ours [16,17].

Pattern of cardiac autonomic dysfunction

Nearly 50% of patients in our study had at least some form of autonomic dysfunction, of which combined autonomic dysfunction was noted in 37% of the patients. A significantly higher prevalence of isolated PNS (28.57 vs. 11.42%), isolated SNS (22.85 vs. 8.57%), and combined autonomic dysfunction (37.14 vs. 2.85%) was found in group 1 compared to group 2 (all p-values <0.05). In a study conducted by Young et al., isolated SNS dysfunction was found in 22.2% of the peripheral neuropathy group, isolated PNS dysfunction was not found in any of the groups, and cardiac autonomic function abnormalities were found in 59% of type 2 diabetics [18]. In the same study, combined sympathetic and parasympathetic dysfunctions were observed in 55.6% of the peripheral neuropathy group which was significantly higher compared to another group [18].

Individual test abnormalities

The most common abnormal PNS test in all of the groups in our study was the E/I ratio on deep breathing, and the most common abnormal SNS test was the diastolic blood pressure change with sustained hand grip. However, a similar study by Naik et al. found that the most common abnormal PNS and SNS tests were the 30:15 ratio on standing and sustained hand grip, respectively [19]. In patients with DPN, there were no statistically significant correlations between the presence of hypertension, the length of diabetes mellitus, HbA1C, lipid profiles, urine microalbumin levels, and sex and cardiac autonomic dysfunction, though the presence of hypertension has been identified as a predictor of cardiac autonomic dysfunction in studies by Witte et al. & Chung et al. [7,20]. In a similar study by Lluch et al., cardiovascular autonomic neuropathy was significantly higher (72.9% vs. 20.6%) in patients with peripheral neuropathy [21]. They discovered a substantial association between cardiac autonomic dysfunction and peripheral neuropathy, independent of age, which is similar to our findings. However, they also discovered an increase in the prevalence of CAN with the length of diabetes, which was not observed in our study. Similar to our findings, however, a study by Vinik et al. in patients with type 2 diabetes mellitus revealed a considerably greater prevalence of CAN in individuals with peripheral neuropathy, regardless of the length of diabetes [22].

Limitations of the study

Our study is a single-center observational study with a lack of follow-up of patients. Evaluation of other aspects of autonomic dysfunction beyond cardiac involvement would have further enhanced our findings. However, ours is the first study to elucidate cardiac autonomic dysfunction in type 2 diabetes mellitus patients with only peripheral neuropathy and no other microvascular complications. As such, this could prove to be instrumental in paving the way for future multicentric research into this aspect.

## Conclusions

Our study, conducted among type 2 diabetes mellitus patients at a southern Indian tertiary care hospital, showed a significantly higher prevalence of cardiac autonomic dysfunction in patients with DPN without other microvascular or macrovascular complications, irrespective of age, sex, or duration of diabetes mellitus. Furthermore, patients with a higher BMI were found to have significantly increased cardiac autonomic dysfunction. Our findings suggest that almost half of the patients with DPN alone have some form of isolated or combined parasympathetic and/or sympathetic autonomic dysfunction. Based on the patterns of autonomic dysfunction seen in our population, screening for increased diastolic pressure on sustained handgrip and variations in the E/I ratio could help identify the most common sympathetic and parasympathetic abnormalities, respectively. Routine implementation of these tests in an outpatient setting could prove to be very useful in the early detection of cardiac autonomic dysfunction, especially in patients with diabetes who have developed peripheral neuropathy complications.
